# Fall patterns and outcomes after a moderate urban earthquake without structural collapse: a retrospective cohort from Istanbul

**DOI:** 10.1186/s40621-026-00699-w

**Published:** 2026-07-08

**Authors:** Emin Uysal, Suleyman Solak, Yahya Acar

**Affiliations:** 1 Department of Emergency Medicine, University of Health Sciences, Bakirkoy Dr. Sadi Konuk Training and Research Hospital, 34147 Istanbul, Turkey; 2 Department of Emergency Medicine, University of Health Sciences, Istanbul Bagcilar Training and Research Hospital, 34200 Istanbul, Turkey; 3 School of Medicine, Department of Pathology and Clinical Sciences Education, Mercer University, Macon, 31201 GA USA

**Keywords:** Earthquakes, Disaster Planning, Urban Population, Public Health, Emergency Service, Hospital, Wounds and Injuries, Behavior-associated injury

## Abstract

**Background:**

Moderate earthquakes — where structural collapse is absent — represent an underexplored public health challenge. In such events, injuries arise not from building failure but from behavior-associated injury mechanisms. This study aims to characterize injury patterns and mechanisms among individuals presenting to a tertiary emergency department following the 6.2 Mw Istanbul earthquake of April 23, 2025, and to identify implications for community-level behavioral preparedness.

**Methods:**

This retrospective study was conducted at a tertiary care hospital serving a catchment population of approximately 750,000 in Istanbul, Turkiye. All individuals presenting to the emergency department within 12 h of the earthquake were included. Cases were identified using the ICD-10 code X34 (earthquake victim). Demographic data, injury mechanisms, anatomical injury sites, need for medical and surgical intervention, and clinical outcomes were recorded. Statistical analysis was performed using IBM SPSS Statistics version 31.0.

**Results:**

A total of 66 individuals were included, representing more than 1/3 of all earthquake-related injuries reported across Istanbul. The mean age was 30.5 ± 13.0 years; 54.5% were male. All recorded injuries were classified as behavior-associated injury mechanisms: same-level falls (W18, 40.9%), falls from stairs (W10, 37.9%), falls or jumps from height (Y30, 12.1%), impact with objects (W22, 6.1%), and entrapment between objects (W23, 3.0%). Injuries coded as W10 and Y30 were significantly more likely to require advanced medical intervention (*p* < 0.001) and surgical treatment (*p* = 0.002). The lower extremity was the most commonly affected region (65.2%). Eleven patients (16.7%) required surgical intervention; no deaths occurred.

**Conclusions:**

In this cohort, all recorded injuries were classified as behavior-associated injury mechanisms. These findings suggest that earthquakes without major structural collapse may represent an important and potentially preventable public health burden.

**Supplementary Information:**

The online version contains supplementary material available at https://doi.org/10.1186/s40621-026-00699-w.

## Introduction

Each year, more than 500,000 earthquakes are recorded worldwide, the vast majority of which are too small or remote to be perceived by humans. However, approximately 3,000 earthquakes are felt by human populations, and 7 to 11 of these events result in significant loss of life [1]. Turkiye is among the countries with the highest seismic risk globally, with most of its territory (84%) located on active fault zones [2]. The 1999 Izmit earthquake and the more recent 2023 Kahramanmaras earthquakes resulted in catastrophic destruction and thousands of deaths, underscoring the enduring public health burden of seismic events in the region. Densely populated metropolitan areas such as Istanbul remain particularly vulnerable to earthquake-related morbidity and mortality.

While research on catastrophic earthquakes is extensive, moderate seismic events in which structural collapse does not occur, represent a distinct and underexplored public health challenge [3]. In such events, injuries are more commonly associated with human behavioral responses than building failure, particularly behavior-associated actions such as running, jumping, or rushing toward exits. Understanding these behavioral injury mechanisms is essential for designing effective community-level preparedness interventions [4].

Istanbul, with a population exceeding 15 million, represents one of the highest-risk urban settings in the world for earthquake exposure. On April 23, 2025, the city experienced a 6.2 Mw earthquake, providing a rare opportunity to examine the public health impact of a moderate urban seismic event in real time. This study aims to characterize injury patterns and mechanisms among individuals presenting to a tertiary emergency department in the acute phase of this earthquake.

## Methods

This retrospective study was conducted at a tertiary care hospital serving a catchment population of approximately 750,000 located in Istanbul, Turkiye, following the earthquake on April 23, 2025.

**Fig. 1:**
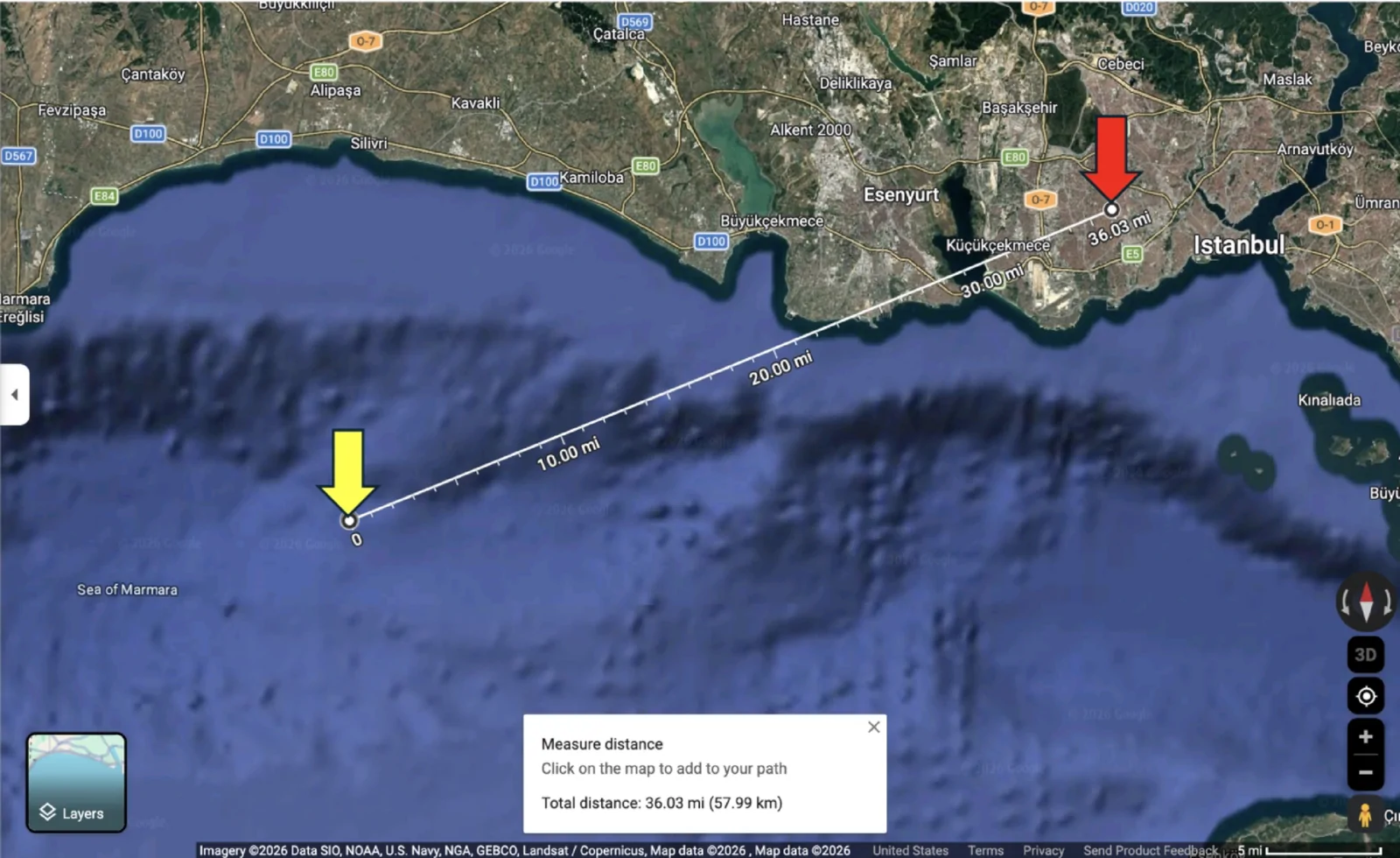
Map showing the distance between epicenter of the earthquake and the hosting institution. (Yellow arrow: earthquake epicenter; red arrow: study hospital; distance: 36.03 miles (57.99 km))

The earthquake magnitude was reported as 6.2 Mw [5], and the epicenter was located approximately 57.99 km (36.03 miles) from the study hospital (Fig. 1). According to official reports, 236 people sustained injuries, of whom 173 were from Istanbul, primarily due to jumping from buildings [5]. Patients who presented to the ED within 12 h of the earthquake were included to represent the acute phase—when major trauma, mortality, and potential chaos are most likely to occur.

Following the earthquake, the hospital activated its disaster and crisis management protocol. Patients presenting with earthquake-related complaints were specifically identified during triage and directed to a designated crisis management team for evaluation. All earthquake-related injury cases underwent standardized clinical assessment and medicolegal documentation in accordance with institutional procedures. As part of this process, ICD-10 code X34 (Earthquake victim) was systematically assigned to earthquake-related presentations, with additional diagnostic codes recorded when appropriate.

Data were collected retrospectively from the hospital’s electronic medical record system. To ensure systematic case identification at the population level, cases were subsequently identified through retrospective review of the electronic medical record using ICD-10 code X34.

Demographic data (age, gender, nationality), mechanism of trauma, anatomical site of injury, need for simple medical intervention, need for surgical intervention, consultations, clinical outcomes, and mortality were recorded. Simple medical intervention was defined as treatment that could be completed in the emergency department without specialty consultation, hospitalization, or surgery. Cases requiring specialist consultation, hospitalization, fracture management, or surgical treatment were categorized as requiring more advanced medical intervention.

### Statistical Analysis

Data analysis was performed using IBM SPSS Statistics version 31.0 (IBM Corp., Armonk, NY, USA). Categorical variables were expressed as frequencies and percentages, while continuous variables were summarized as mean ± standard deviation (SD) or median with interquartile range (IQR), depending on data distribution. The Kolmogorov–Smirnov test was used to assess the normality of continuous variables. Comparisons of continuous variables between independent groups were performed using the Student’s t-test. The chi-square test was used for categorical variables. A p-value < 0.05 was considered statistically significant.

The local institutional review board (IRB) approved the study protocol (Approval date: May 22, 2025; Decision No: 2025/05/06/049) and the authors followed the principles of the Declaration of Helsinki at all steps.

## Results

A total of 66 earthquake-injured individuals were included, representing more than 1/3 (66/173) of all earthquake-related injuries reported across Istanbul [5]. The mean age was 30.5 ± 13.0 years (min: 1, max: 57), with 36 males (54.5%) and 30 females (45.5%). Most patients were Turkish citizens (*n* = 58, 87.9%), while 8 (12.1%) were foreign nationals.

A total of 32 patients (48.5%) presented within the first 6 h of the earthquake, while 34 (51.5%) presented between 6 and 12 h (Table 1). Most patients arrived as walk-ins (*n* = 57, 86.4%), whereas 9 (13.6%) arrived by ambulance. There was no difference between walk-in and ambulance arrivals regarding admission time (*p* = 0.794) or need for surgery (*p* = 0.630). A significant difference was observed between males (33.3% vs. 21.2%) and females (15.2% vs. 30.3%) for first six-hour admissions (*p* = 0.025) (Table 1).

**Table 1:** Demographics of the study population and comparison between early and late emergency department admissions

		First 6 h	6–12 h	Total	p
Age		30.3±14.5 (min:1, max:57)	30.7±11.7 (min:4, max:55)	30.5±13.0 (min:1, max:57)	0.911*
Gender	Female	10 (15.2%)	20 (30.3%)	30 (45.5%)	0.025**
	Male	22 (33.3%)	14 (21.2%)	36 (54.5%)	
Simple medical intervention	Manageable	17 (25.8%)	22 (33.3%)	39 (59.1%)	0.339**
	Not manageable	15 (22.7%)	12 (18.2%)	27 (40.9%)	
Surgery	Negative	24 (36.4%)	31 (47.0%)	55 (83.3%)	0.078**
	Positive	8 (12.1%)	3 (4.5%)	11 (16.7%)	

*Student’s t-test

**Chi-square test

Percentages were calculated using the total study population (*n* = 66)

The most common injury mechanism was “fall on the same level, other or unspecified” (ICD-10: W18), followed by “fall on stairs or steps” (ICD-10: W10) and “fall from height/jumping” (ICD-10: Y30) (Table 2). Injuries coded as W10 and Y30 were significantly more likely to require advanced medical interventions (*p* < 0.001) and surgical treatment (*p* = 0.002) (Table 2). Among the eight fall-from-height/jumping cases, all were Turkish citizens, most were male (62.5%), seven (87.5%) presented within the first 6 h, and five (62.5%) required surgery.

**Table 2:** The comparison of the trauma mechanisms and outcomes

Trauma mechanism (ICD-10 code)	n (%)	Simple medical intervention			Surgery		
		Manageable	Not manageable	p*	Negative	Positive	p*
Fall on or from stairs and steps (W10)	25 (37.9)	10 (15.2%)	15 (22.7%)	**< 0.001**	20 (30.0%)	5 (7.6%)	**0.002**
Falling, jumping or pushed from a high place, undetermined intent (Y30)	8 (12.1)	2 (3.0%)	6 (9.1%)		3 (4.5%)	5 (7.6%)	
Striking against or struck by other objects (W22)	4 (6.1)	4 (6.0%)	0 (0%)		4 (6.0%)	0 (0%)	
Caught, crushed, jammed or pinched in or between objects (W23)	2 (3.0)	0 (0%)	2 (3.0%)		2 (3.0%)	0 (0%)	
Fall on the same level, other or unspecified (W18)	27 (40.9)	23 (34.9%)	4 (6.0%)		26 (39.4%)	1 (1.5%)	

*Chi-square test

The most commonly affected anatomical region was the lower extremity (*n* = 43, 65.2%), followed by the upper extremity (*n* = 13, 19.7%). Multiple anatomical injury sites were documented in one patient (1.5%). The most frequent clinical findings were tenderness (*n* = 38, 57.6%) and fracture (*n* = 27, 40.9%). No psychiatric complaints were documented during emergency department presentation.

The mean age of patients requiring only simple medical intervention was 27.3 ± 12.5 years, compared to 35.2 ± 12.5 years among those requiring more advanced care (*p* = 0.014).

More than half of the patients (*n* = 36, 54.5%) required no specialty consultation. Orthopedic consultations were requested for 25 patients (37.9%). Surgical intervention was required in 11 patients (16.7%), whereas 55 (83.3%) were managed non-operatively.

Most patients (*n* = 52, 78.8%) were discharged home, 11 (16.7%) were hospitalized, 1 (1.5%) was transferred to another facility due to maxillofacial trauma, and 2 (3.0%) left against medical advice. No deaths occurred.

## Discussion

This study investigated the public health impact of the 6.2 Mw Istanbul earthquake on an urban population, with a focus on behavioral injury mechanisms and their implications for community-level preparedness. Unlike catastrophic events such as the 1999 Izmit and 2023 Kahramanmaras earthquakes, no structural collapse occurred. Nevertheless, a substantial number of injuries were recorded and were classified as behavior-associated injury mechanisms — including falls on the same level, falls from stairs, and jumps from height. These findings suggest that moderate earthquakes without major structural collapse may still represent an important public health burden, with injuries potentially arising from behavioral responses rather than structural vulnerability. To the best of our knowledge, this is the first study to characterize behavior-associated injury mechanisms following a moderate earthquake in an urban Turkish population.

The observation that all recorded injuries in this cohort were classified as behavior-associated injury mechanisms rather than building collapse may have implications for public health intervention. Behavioral responses during an earthquake are not merely individual phenomena; they may also reflect broader gaps in community preparedness that can be addressed through targeted education and training. Our findings align with Shoaf et al., who reported that approximately 15% of earthquake-related injuries in California resulted from inappropriate reactions such as jumping from windows or attempting to catch falling objects [6]. The consistency of these patterns across geographically and culturally distinct populations suggests that behavior-associated injury mechanisms may occur across different settings and warrant further investigation.

Literature specifically addressing non-destructive earthquakes remains limited, underscoring the public health relevance of the present study. Yildirim et al. reported 275 emergency department admissions following the 2014 Gokceada earthquake (6.5 Mw), with injuries predominantly involving soft-tissue damage and fractures [7]. Keskin et al. found that after the 1999 Golcuk earthquake, 70.4% of admissions involved minor injuries [8]. In our cohort, 48.5% of individuals presented within the first six hours — a proportion somewhat lower than previously reported, likely reflecting the impact of traffic congestion and public health messaging discouraging vehicle use in the immediate aftermath. This observation itself points to the effectiveness of real-time public communication as a public health tool during seismic events.

Studies from destructive earthquakes consistently document extremity injuries but attribute them primarily to crush injuries and structural collapse [9–12]. Eyler et al., analyzing admissions following the 2020 Izmir earthquake (6.9 Mw), reported 68.7% soft-tissue injuries and 21.7% fractures without detailing the underlying mechanisms [13]. The present study contributes to literature by describing specific behavioral mechanisms associated with injuries following an urban earthquake in the absence of major structural collapse — a distinction critical for designing prevention strategies that target behavior rather than infrastructure.

Public health preparedness programs in Turkiye have long sought to promote appropriate earthquake responses. Celikmen et al. noted that the “triangle of life” position was more commonly adopted during the 2023 Kahramanmaras earthquakes compared to the 1999 Marmara earthquake, suggesting some progress in public awareness [14]. However, the “triangle of life” technique remains controversial and is not evidence-based. Globally endorsed strategies such as “Drop, Cover, and Hold On” are scientifically supported and have been shown to reduce injury risk [15]. The persistence of reactive behaviors in our study population — despite decades of public awareness efforts — may suggest that knowledge alone is insufficient, and that behavioral preparedness programs might be redesigned to foster automatic protective responses under stress.

Achieving this requires a broader public health approach. Muttarak et al. and Shapira et al. have demonstrated that education, socioeconomic status, housing type, and prior earthquake experience are key determinants of behavioral response [16, 17]. These findings point to the need for differentiated preparedness strategies that account for population heterogeneity. In high-density urban settings such as Istanbul, where millions of residents face significant seismic exposure, integrating behavioral preparedness into school curricula, workplace safety programs, and community drills may contribute to reducing the preventable injury burden associated with moderate earthquakes.

### Limitations

The current study was conducted at a single center, which may limit generalizability. The relatively small sample size and descriptive study design limited more advanced statistical analyses; therefore, the findings should be interpreted as descriptive rather than causal. The study did not collect information regarding prior earthquake training, preparedness knowledge, or adherence to specific protective strategies, limiting our ability to evaluate the relationship between preparedness behaviors and injury outcomes. Psychiatric symptoms were not systematically assessed, and acute psychological distress may have been underrecognized or incompletely documented during emergency department evaluation. Although earthquake-related patients were managed through a dedicated disaster response pathway, retrospective identification based on ICD-10 coding may have resulted in occasional misclassification or under-ascertainment of eligible cases. Additionally, data on patients’ educational level, socioeconomic status, or previous earthquake experience were unavailable, preventing deeper behavioral analysis.

## Conclusion

In this cohort, all recorded injuries were classified as behavior-associated injury mechanisms—including falls on the same level, falls from stairs, and jumps from height. These findings suggest that earthquakes without major structural collapse may represent an important and potentially preventable public health challenge.

The persistence of behavior-associated injuries in a major metropolitan area with established public awareness programs may reflect a gap between public awareness efforts and observed protective behavior. Our findings support consideration of preparedness approaches that extend beyond information dissemination alone and include interventions that build automatic, stress-resilient responses — through regular drills, school-based education, and workplace safety programs. Given that the injuries observed in this study were associated with behavioral responses, further efforts to improve earthquake preparedness and protective behaviors may be warranted. Existing international preparedness recommendations, including “Drop, Cover, and Hold On,” may provide a useful framework for such initiatives.

In high-density urban settings such as Istanbul, where seismic risk is both high and well-documented, integrating behavioral preparedness into broader public health policy may help reduce the burden of preventable earthquake-related injuries. Future studies incorporating data on prior earthquake experience, socioeconomic status, and educational background would further strengthen the evidence base for targeted community interventions.

## Supplementary Information

Supplementary Material 1

## Data Availability

The dataset supporting the conclusions of this article is included as Additional file 1.

## Abbreviations

ED: Emergency department
ICD-10: International Classification of Diseases, 10th revision
IQR: Interquartile range
IRB: Institutional review board
SD: Standard deviation
SPSS: Statistical Package for the Social Sciences
